# Efficacy of prostaglandina E1 for the treatment of patients with thrombo-occlusive vasculitis: A protocol of systematic review and meta-analysis

**DOI:** 10.1097/MD.0000000000020369

**Published:** 2020-05-29

**Authors:** Yao Sun, Han-rui Wang, Xiao-hang Feng, Kai-feng Wang

**Affiliations:** a Department of Vascular Surgery; b Department of Otorhinolaryngology Surgery, First Affiliated Hospital of Jiamusi University, Jiamusi, China.

**Keywords:** efficacy, prostaglandina E1, thrombo-occlusive vasculitis

## Abstract

**Background:**

This study aims to explore the efficacy and safety of prostaglandina E1 (PE1) for the treatment of patients with thrombo-occlusive vasculitis (TOV).

**Methods:**

Electronic databases (Cochrane Library, PUBMED, EMBASE, Web of Science, Scopus, the Allied and Complementary Medicine Database, Chinese Biomedical Literature Database, and China National Knowledge Infrastructure) will be sought from onset to the March 1, 2020 without language and publication status restrictions. We will include any potential randomized controlled trials that examined the efficacy of PE1 for the treatment of patients with TOV. We will appraise study quality using Cochrane risk of bias tool, and will assess the evidence quality using Grading of Recommendations Assessment Development and Evaluation. We will use RevMan 5.3 Software for statistical analysis.

**Results:**

A high-quality synthesis of present evidence of PE1 for the treatment of patients with TOV will be provided in this study.

**Conclusion:**

This study will provide evidence to judge whether PE1 is an effective intervention for TOV.

**Systematic review registration:**

INPLASY202040081.

## 1 Introduction

Thrombo-occlusive vasculitis (TOV) is a common clinical systemic vasculitis, which is associated with significant morbidity and mortality.^[1–3]^ It is characterized by the segmental, non-suppurative inflammation and intra-arterial thrombosis in small and medium arteriovenous veins.^[4–6]^ This disorder often manifests as ulcerations in oral, genital, skin lesions, vascular, neurological and gastrointestinal tissues.^[7–9]^ Although it has been recognized for a few decades, its mechanisms are still poorly understood.^[4,10]^

Prostaglandina E1 (PE1) has been extensively utilized for the treatment of patients with TOV.^[11–21]^ However, no systematic review assessed the efficacy and safety of PE1 for TOV. Thus, this study will carry out a systematic review to evaluate the strength of the present evidence to support the efficacy and safety of PE1 for the treatment of TOV.

## 2 Methods and analysis

### 2.1 Study registration

This study protocol has been registered through INPLASY202040081. It is reported according to the guideline of Preferred Reporting Items for Systematic Reviews and Meta-Analysis Protocol statement.^[22]^

### 2.2 Dissemination and ethics

This study will be published via a peer-reviewed journal or a related conference. This study will not need ethic approval, since no individual data will be extracted.

### 2.3 Eligibility criteria

#### 2.3.1 Participants/subjects

We will include patients who were diagnosed as TOV, in spite of their race, age, gender, and severity of TOV.

#### 2.3.2 Interventions/exposure

In the experimental group, all patients received PE1 for their solely treatment will be included.

In the control group, there are no limitations to any comparators, such as herbal medicine. However, we will exclude studies which involved PE1 as their controls.

#### 2.3.3 Study types

This study will include randomized controlled trials (RCTs), which explored the efficacy and safety of PE1 for the treatment of patients with TOV.

#### 2.3.4 Outcome measurements

Primary outcomes include hemorheological indicators (such as blood fluidity, viscosity, deformability, and coagulation of high-cut blood viscosity, low-cut blood viscosity, plasma specific viscosity, and red cell aggregation index).

Secondary outcomes consist of skin temperature of affected limb, ankle brachial index, painless walking distance, maximum walking distance, ulcer area of the affected limb, serological indicators, recurrence rate, incidence of complications (such as infection, coagulopathy, secondary thrombosis) and amputation rate.

### 2.4 Literature search

The following electronic databases (Cochrane Library, PUBMED, EMBASE, Web of Science, Scopus, the Allied and Complementary Medicine Database, Chinese Biomedical Literature Database, and China National Knowledge Infrastructure) will be sought from commencement to the March 1, 2020 with no limitations to language and publication status. All potential RCTs that investigated the efficacy of PE1 for the treatment of patients with TOV will be included. Search strategy sample for Cochrane Library will be presented (Table 1). We will adapt equivalent search strategies for other electronic databases.

**Table 1:**
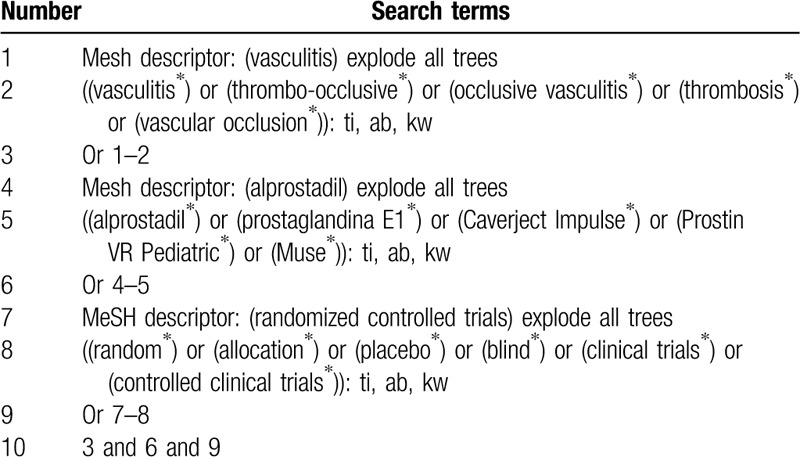
Search strategy of Cochrane library.

We will seek secondary literature sources to avoid missing potential studies, such as Google scholar, conference abstracts, and reference lists of relevant reviews.

### 2.5 Literature selection

Two reviewers will separately screen titles and abstracts of all identified to remove all irrelevant studies. Then, we will obtain full papers of potential articles according to the eligibility criteria. Finally, we will include all eligible studies. Any conflicts between 2 reviewers will be cleared up by a third reviewer through discussion. We will summarize the results of study selection in a preferred reporting items for systematic reviews and meta-analysis flow chart.

### 2.6 Data extraction and management

Two reviewers will separately perform data extraction using pre-constructed data extraction sheet. Different opinions will be solved by discussion with the help of a third reviewer. The extracted information consists of general trial information (eg, first author, year of publication), baseline patient characteristics (eg, gender, age, disease severity and duration), trial methods (eg, randomization, blind), details of intervention and controls, outcomes, adverse events, and other related information.

### 2.7 Risk of bias assessment

Two reviewers will independently evaluate the risk of bias of all eligible RCTs using Cochrane risk of bias tool. It comprises of 7 domains and each item is graded as high, unclear, or low risk of bias. Any differences will be solved by discussion with the help of a third reviewer.

### 2.8 Statistical analysis

#### 2.8.1 Data synthesis

This study will use RevMan 5.3 software for statistical analysis. To summarize the effects of PE1 treatment for each trial, weighted mean difference or standardized mean difference and 95% confidence intervals will be utilized when the result is continuous data. In case of dichotomous data, risk ratio and 95% confidence intervals will be exerted. *I*^2^ test is utilized to check statistical heterogeneity. A fixed-effects model will be employed if there is homogeneity across the data (*I*^2^ ≤50%), and a random-effects model will be applied if obvious heterogeneity is present (*I*^2^ > 50%). If necessary, we will carry out a meta-analysis. Otherwise, we will perform a subgroup analysis if considerable heterogeneity is found.

#### 2.8.2 Subgroup analysis

Subgroup analysis will be utilized to investigate the sources of heterogeneity based on the different treatments, controls, and outcomes.

#### 2.8.3 Sensitivity analysis

Sensitivity analysis will be required to test the robustness of conclusions by removing low quality studies.

#### 2.8.4 Reporting bias

A funnel plot and Egger regression test will be conducted to examine reporting bias if more than10 trials are included.^[23]^

### 2.9 Grading the quality of evidence

We will check the evidence level for major outcomes by 2 independent reviewers using Grading of Recommendations Assessment Development and Evaluation.^[24]^ Any disagreements will be solved by a third reviewer through discussion to reach a consensus.

## 3 Discussion

TOV is 1 of the most severe systemic vasculitis disorders. If no effective treatment is administrated, it may lead to high mortality and morbidity. To our best knowledge, this study will firstly evaluate the efficacy and safety of PE1 for the treatment of TOV. It will retrieve as comprehensive literature sources as possible without language limitations. All potential RCTs regarding the PE1 for the treatment of TOV will be fully considered. The results of this study may provide most recent evidence on the efficacy and safety of PE1 for the treatment of TOV.

## Acknowledgments

This work has supported by the Heilongjiang Provincial Department of Education Fundamental Research Project for Basic Scientific Research Expenses (2016-KYYWF-0582). The financial supporter will not participate any parts of this study.

## Author contributions

**Conceptualization:** Yao Sun, Han-rui Wang, Xiao-hang Feng, Kai-feng Wang.

**Data curation:** Yao Sun, Han-rui Wang, Kai-feng Wang.

**Formal analysis:** Yao Sun, Xiao-hang Feng, Kai-feng Wang.

**Investigation:** Kai-feng Wang.

**Methodology:** Yao Sun, Han-rui Wang, Xiao-hang Feng.

**Project administration:** Kai-feng Wang.

**Resources:** Yao Sun, Han-rui Wang, Xiao-hang Feng.

**Software:** Yao Sun, Han-rui Wang, Xiao-hang Feng.

**Supervision:** Kai-feng Wang.

**Validation:** Yao Sun, Han-rui Wang, Xiao-hang Feng, Kai-feng Wang.

**Visualization:** Yao Sun, Kai-feng Wang.

**Writing – original draft:** Yao Sun, Han-rui Wang, Xiao-hang Feng, Kai-feng Wang.

**Writing – review & editing:** Yao Sun, Xiao-hang Feng, Kai-feng Wang.
